# The first epidemiological and virological influenza surveillance in the Republic of Guinea revealed the predominance of influenza A/H3N2 and B Victoria viruses

**DOI:** 10.1017/S0950268821001965

**Published:** 2021-09-28

**Authors:** Mamadou Bhoye Keita, Fenano Pierre, Jean Ndjomou, Bassala Traoré, Pepe Tohonamou, Mafoudia Soumaré, Sidibé Mamadi, Moussa Aminata Keita, Célestin Ebi Bile, Raymond Bernard Pallawo, Soatiana Cathycia Rajatonirina, Ahmadou Barry, Lamine Koivogui, Robert Camara, Abdoulaye Touré

**Affiliations:** 1 Institut National de Santé Publique (INSP), PO Box 6623, Conakry, Guinea; 2 Université Gamal Abdel Nasser Conakry, Guinea; 3 MRIGlobal, 65 W. Watkins Rd, Gaithersburg, MD 20878, USA; 4 US Centers for Disease Control and Prevention, Atlanta, GA, USA; 5 World Health Organization, Conakry, Guinea; 6 World Health Organization, Brazzaville, Congo

**Keywords:** A/H3N2, B/Victoria, Guinea, influenza, surveillance, typing

## Abstract

Little is known about respiratory viruses infection in Guinea. Influenza surveillance has not been implemented in Guinea mainly because of the paucity of laboratory infrastructure and capacity. This paper presents the first influenza surveillance data in Guinea.

Swabs were obtained from August 2018 through December 2019 at influenza sentinel sites and transported to the Institut National de Santé Publique for testing. Ribonucleic acid was extracted and tested for the presence of influenza A and B by real-time reverse transcription-polymerase chain reaction (RT-PCR). Positive samples were further characterised to determine the subtypes and lineages of influenza viruses.

A total of 862 swabs were collected and tested. Twenty-three per cent of samples tested positive for influenza A and B viruses. Characterisation of positive specimens identified influenza A/H1N1pmd09 (2.5%), influenza A/H3N2 (57.3%), influenza B/Victoria lineage (36.7%) and 7 (3.5%) influenza B with undetermined lineage. Influenza B virus activity clustered in August through November while influenza A/H3N2 displayed two clusters of activities that appeared in May through August and November through December.

For the first time in Guinea, the epidemiology, diversity and period of circulation of influenza viruses were studied. The results indicate the predominance and the periods of activities of influenza B Victoria lineage and influenza A/H3N2 which are important information for preventive strategies. It is warranted to extend the influenza surveillance to other parts of Guinea to better understand the epidemiology of the viruses and monitor the emergence of influenza strains with pandemic potential.

## Background

Influenza is an acute respiratory infection due to influenza viruses types A and B. Transmission occurs through droplets containing viruses that spread in the air during coughing and sneezing. Global annual deaths due to influenza-associated respiratory infections are estimated at 650 000, most of which are in sub-Saharan Africa [1]. Influenza-associated hospitalisation in children <5 is three times higher in Africa than in industrialised countries [2]. Although influenza viruses cause illness to all age groups, children <5 and adults ⩾65 years old are mostly affected [2–4]. It has also been shown that pathologies such as HIV/AIDS and tuberculosis exacerbate the risk and severity of influenza disease [5–7].

The epidemiology of influenza in industrialised countries is well characterised and provides sufficient information for influenza vaccine updates. However, in sub-Saharan Africa, there is a paucity in the knowledge of epidemiology and the burden of morbidity due to influenza. In West Africa, influenza surveillance systems are becoming increasingly strengthened, with several countries now having national influenza surveillance systems. These surveillance systems have provided important information on the seasonality and circulation of influenza viruses in West Africa [8–15]. However, this has not been the case for the Republic of Guinea, mostly because of the paucity and weakness of the laboratory system.

According to data from WHO country profile, lower respiratory infections represent the first cause of mortality in Guinea, accounting for 12.5% of total deaths far ahead of malaria (10%) and diarrhoeal diseases (6.1%) [16]. From 2014 to 2016, the Republic of Guinea experienced an unprecedented Ebola virus disease outbreak that caused an estimated 2500 deaths [17] and US$ 600 million in economic losses [18]. In response, Guinea' Ministry of Health, with financial support from international partners, and technical support from WHO, has prioritised the early detection of epidemic- and pandemic-prone diseases. Laboratory capacity has been strengthened and improved, including the national influenza reference laboratory. This enabled the initiation of influenza surveillance to inform public health decisions. These new diagnostic and surveillance abilities will allow the Republic of Guinea to readily react to disease threats in the future by gathering data to inform public health decisions.

This study aimed to determine the rate of influenza viruses among patients presenting with respiratory infections and to characterise the circulating influenza virus subtypes and their seasonality.

## Methods

### Study design and surveillance system

We conducted a descriptive study using surveillance data from August 2018 (week 33) through December 2019 (week 52). The established surveillance system was based on WHO' integrated disease surveillance and response (IDSR) regional generic protocol for influenza sentinel surveillance and was developed according to the WHO/AFRO (Africa) training manual on influenza surveillance [19]. The system included an epidemiology unit with a focal point responsible for the coordination, supervision and data entry for each of the sentinel sites and the National Influenza Laboratory (NIL) responsible for sample testing and data analysis. Healthcare workers, physicians, nurses and laboratorians at the sentinel sites were trained on the use of standardised case definitions of influenza-like illness (ILI) and severe acute respiratory infection (SARI) cases. Laboratorians at the sentinel sites also received training on nasopharyngeal and oropharyngeal swab sample collection, storage and transportation.

### Surveillance sites

Sentinel sites included four health centres located in four communes in Conakry, the capital of the Republic of Guinea. The health centre of Maciré in the Dixinn commune (GPS coordinates: 9.549689; −13.665889), the health centre of Koulewondy in the commune of Kaloum (GPS coordinates: 9.508638; −13.704817) and the health centre of Gbessia Port in the commune of Matoto (GPS coordinates: 9.561002; −13.627166) were ILI sentinel sites. The fourth site was the Communal Medical Center (CMC) of Ratoma in the Ratoma commune (GPS coordinates: 9.583781; −13.658753) and represented the SARI sentinel site.

### Study population

Suspected ILI and SARI cases were screened according to the WHO case definition of ILI and SARI [4, 20]. Thus, any outpatient with an acute respiratory illness with temperature ⩾38 °C and cough, within 10 days of symptoms onset, was eligible for ILI enrolment. SARI was considered as an acute respiratory infection with a measured or history of fever ⩾38 °C and cough, with the onset of symptoms within the past 10 days that required hospitalisation.

### Sample collection

A structured questionnaire was used to collect the socio-demographic and clinical data on each enrolled patient. The nasopharyngeal and oropharyngeal swabs were obtained at each sentinel site from the first five patients meeting the inclusion criteria. After collection, swabs were immediately inserted into a tube containing the viral transport medium (VTM) (Becton Dickinson, US) and then refrigerated. At the end of the day, collected samples were transported to the NIL at the Institut National de Santé Publique (INSP). Upon arrival at the laboratory, tubes containing the sample were vortexed for 30 s and three aliquots were made for each sample and stored at −20 °C. One aliquot was used for RNA extraction.

### Nucleic acid extraction and testing

Viral RNA was extracted from 200 μl of the specimen' aliquot using the QIAamp viral mini RNA kit (Qiagen, Germany) following the manufacturer' instructions. RNA was eluted in 100 μl of RNase/DNase-free elution buffer provided with the kit. Extracted RNA was analysed for the presence of influenza viruses by real-time reverse transcription-polymerase chain reaction (RT-PCR) on the ABI 7500 Fast Dx Real-time PCR instrument (Applied Biosystems, US). The reagents, primers and probes used for influenza viruses' detection and subtyping were from the US Centers for Disease Control and Prevention (US CDC). To assess the quality of each sample, the human ribonuclease P gene was amplified from all samples tested. All real-time RT-PCR data were analysed using the ABI sequence detection software version 1.4 (Applied Biosystems, US).

### Data analysis

Socio-demographic and clinical data obtained on patients were recorded in an Excel spreadsheet (Microsoft Office 2010). For the analyses of ILIs, we calculated the proportion of ILI in each age group per total number of consultations. For confirmed influenza cases analysis, the rate of positivity was calculated for each age group per total number of influenza confirmed cases. The difference between categories was examined using the chi-square test. For the analysis of the period of the circulating virus, positive samples were linked to the corresponding week, then month and year. The predominance of a virus subtype is defined as the higher proportion or number of that virus overall viruses detected during a specific period of time.

## Results

### Clinical manifestations and characteristics of the study population

A total of 10 599 patients visited the sentinel sites during the study period. One third (the majority) of the patients visiting the health centres aged between 15 and 50 years old. Of all patients who visited the health centres, 30.2% had ILI symptoms. Patients aged between 2 and 5 years accounted for the majority (48.3%) of those with ILI symptoms. This group was followed by those aged less than 2 years (41%). Patients over 65 years old represented the least (9%) of those with ILI symptoms (Table 1). Fifty-three per cent of the enrolled population were female and 46.8% were male (Table 2).

**Table 1.** Proportion of ILI over total consultations

Consultations	Age range (Year)						Total
	0–<2	2–<5	5–<15	15–<50	50–<65	⩾65	
All illness (*n*)	2497	1846	2510	3384	273	89	10 599
ILI (*n*)	1024	891	755	489	29	8	3196
Proportion ILI (%)	41.0	48.3	30.1	14.5	10.6	9.0	30.2

**Table 2.** Proportion of influenza-positive by demographic characteristics

Demographic characteristics	Number *n* (%)			95% CI^a^ positive
	Total	Negative	Positive	
**Sex**				
Male	403 (46.8)	315	88 (21.8)	17.9–16.2
Female	459 (53.2)	348	111 (24.2)	20.3–28.4
**Age**				
<2	270 (31.3)	225	45 (16.7)	12.6–22.0
2–<5	241 (28.0)	167	74 (30.7)	25.4–37.5
5–<15	146 (16.9)	109	37 (25.3)	18.0–32.7
15–<50	184 (21.3)	142	42 (22.8)	17.0–29.6
50–<65	13 (1.5)	13	0	0
⩾65	8 (0.9)	7	1 (12.5)	0.3–52.7
**Sentinel site**				
Maciré	336 (39.0)	257	79 (23.5)	19.3–28.7
Koulewondy	316 (36.7)	241	75 (23.7)	19.2–28.8
Gbessia port I	191 (22.2)	149	42 (22.0)	16.3–28.5
Ratoma	18 (2.1)	16	2 (11.1)	1.4–34.7
**Total**	862 (100)	663	199 (23.1)	20.3–26.1

a CI is confidence interval of positive.

### Influenza positivity in the study population

Influenza viral RNA was detected in 199 out of 862 swab samples yielding an overall influenza positivity rate of 23.1% (95% CI 20.3–26.1) in the study population. Influenza positivity was 21.8% (95% CI 16.2–17.9) in men and 24.2% (95% CI 20.3–28.4) in women. However, statistical analysis showed that there was no significant difference in influenza positivity with regard to gender (*P* = 0.414; *P* > 0.05). We further calculated the influenza positivity rate according to influenza sentinel sites. The results showed that the influenza positivity rate was 23.5% (95% CI 19.3–28.7) for the Health Center of Maciré, 23.7% (95% CI 19.2–28.8) for the Health Center of Koulewondy, 22% (95% CI 16.3–28.5) for the Health Center of Gbessia Port and 11.1% (95% CI 1.4–34.7) for the Communal Medical Center of Ratoma. Statistical analysis showed there was no significant difference in influenza positivity according to the health centre (*P* = 0.638; *P* > 0.05). We then looked at the influenza positivity in the different age groups and found rates of 30.7% in people aged between 2 and 5 years old, 25.3% in those aged between 5 and 15 years old, 22.8% in people aged between 15 and 50 years old and 16.7% in those under 2 years old (Table 2). Analysis of influenza positivity according to the age group revealed a statistically significant difference (*P* = 0.0009; *P* < 0.01).

### Influenza virus characterisation

Among influenza-positive samples, 59.8% (119/199) were characterised as influenza A virus and 40.2% (80/199) as influenza B viruses. Influenza A positive samples were further subtyped to determine the subtype of the circulating virus. The results showed that 95.8% (114/119) of influenza A virus cases were classified as influenza A subtype H3N2, while the remaining five (4.2%) belonged to the influenza A subtype H1N1pmd09 pandemic. Further characterisation of positive influenza B viruses demonstrated that influenza B Victoria virus accounted for 91.2% (73/80) of all influenza B positive samples. Seven (8.8%) of the influenza B viruses could not be assigned to either Victoria or Yamagata lineage and were therefore classified as undetermined. Overall, among all influenza viruses identified in this study, influenza A subtype H3N2 was the predominant circulating virus accounting for 57.3%. This was followed by influenza B Victoria lineage (36.7%), the undetermined influenza B viruses accounted for 3.5%, and the influenza A subtype H1N1 pdm09 for 2.5% (Table 3). Influenza A and B viruses were distributed among all age groups (Table 3). Four of the seven influenza B viruses with undetermined lineage were identified among children under 5 years old and the remaining three were found in those aged between 5 and 50 years. No influenza B Yamagata lineage was found in the study population (Table 3).

**Table 3.** Distribution of influenza virus types by demographic characteristics

Demographic characteristics	Influenza virus types *n* (%)				
	A/H1N1 pdm09	A/H3N2	B/Victoria	B/Yamagata	B/not groupable
**Sex**					
Male	3	52	30	0	3
Female	2	62	43	0	4
**Age**					
<2	1	31	12	0	1
2–<5	1	41	29	0	3
5–<15	2	24	10	0	1
15–<50	1	17	22	0	2
50–<65	0	0	0	0	0
⩾65	0	1	0	0	0
**Sentinel site**					
Maciré	2	41	37	0	0
Koulewondy	3	42	27	0	3
Gbessia port I	0	29	9	0	4
Ratoma	0	2	0	0	0
**Total**	5 (2.5)	114 (57.3)	73 (36.7)	0	7 (3.5)

### Period of influenza viruses' activity

**Fig. 1.**
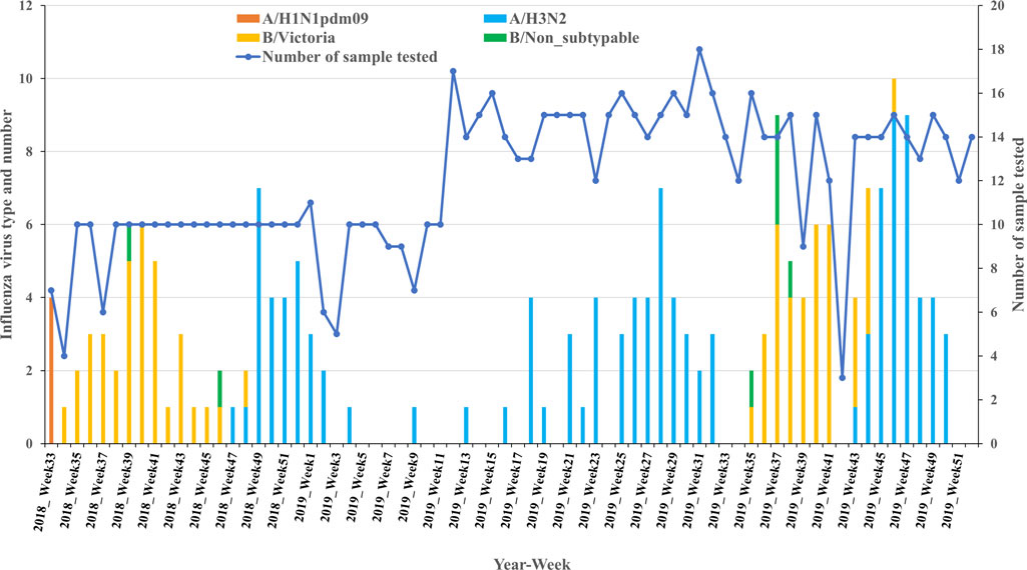
Weekly distribution of subtypes and lineages of confirmed influenza A and B viruses during 2018 and 2019.

We monitored influenza viruses from August 2018 through December 2019 to map the period of the year that corresponds to the circulation of each virus. We observed patterns in the circulation of the two predominant influenza viruses. Influenza B virus activity clustered in August through November (weeks 34 through 48) of 2018, depicting a peak of activity at weeks 39 and 40. During 2019, the influenza B virus showed activities again in August through November (week 35 through 46) with peak activity observed at week 37 (Fig. 1). The seasonal influenza A/H3N2 strain displayed two major clusters. The first cluster of activity appeared in November through December of 2018 (week 47 through 52) with peak activity observed at week 49. During 2019, this cluster of influenza A/H3N2 virus was observed from October through December (week 43 through 50) with a peak of activity observed at weeks 46 and 47. The second major cluster of the activity of influenza A/H3N2 virus was observed from May through early August (week 18 through 32), depicting a peak of activity at weak 28 (Fig. 1). In 2019, influenza A/H3N2 virus activity continued through January (week 4) followed by some sporadic cases observed up to April (week18). The pandemic influenza A/H1N1pdm09 showed some appearances at week 33 of 2018 but was not observed during the 2019 year (Fig. 1). Influenza B virus co-circulated with influenza A virus during October (week 43) through early November (week 46) (Fig. 1).

## Discussion

The positivity rate of the influenza viruses (influenza A and B) was estimated at 23.1% in the study population. This rate is consistent with previous influenza surveillance studies conducted in West African countries [9, 11–14]. When influenza positivity was compared across different age groups, a statistically significant difference was found. This finding is in accordance with previous studies revealing an increased influenza positivity in children <5 years old [2, 3].

We found that the epidemiology of influenza viruses was dominated by the circulation of the seasonal influenza A/H3N2 strain and the influenza B Victoria lineage both of which represented 94% of all influenza viruses. These findings are in line with previous influenza surveillance studies reported in sub-Saharan Africa [8, 11, 12, 21, 22]. In contrast to a previous surveillance study conducted in Burkina Faso that reported the circulation of influenza B Yamagata lineage [14], this lineage was not detected in our study. We identified seven influenza B strains that could not be assigned to either the Victoria or Yamagata lineages. The failure to determine their lineage could be explained by the fact that they may represent new variants within the influenza B virus that could not be detected by the sets of primers and probes used in our study. In fact, mutations within the primers or probes binding sites used for differentiation of influenza B viruses into Yamagata and Victoria could lead to the failure of primers or probes to bind to the target sequences. Thus, future characterisation of these undetermined viruses is of paramount importance as they may represent outlier variants within influenza B viruses. Sequencing of these undetermined influenza B viruses will help determining the extent of their sequence variability and comparison with known influenza B Yamagata and Victoria viruses will determine whether they represent new variants/subgroups within influenza B viruses.

The most striking finding was the cluster of the activities of the major circulating influenza viruses. The two predominant influenza viruses (A/H3N2 and B/Victoria) noticeably distinguished each other with regard to the periods of their activity. Influenza B viruses exhibited clear and intense activities during the period of August through November of 2018 that matched with the period of activities in the subsequent year of 2019. This period of activity of the influenza B virus corresponds to the rainy season in Conakry. The seasonal influenza A/H3N2 displayed two major patterns of activities during the year. The first cluster of activity of influenza A/H3N2 started in May through early August with peak activity in July at week 28. The second cluster of activity was observed from late November through December with peak activity at weeks 46–47. Both clusters of intense activities of A/H3N2 correspond to the rainy season in Conakry. Some sporadic, co-circulation of influenza A/H3N2 and influenza B Victoria lineage were observed in late October−early November (week 43–46). However, this could be due to the overlapping of the end of influenza B Victoria activity and the onset of the seasonal influenza A/H3N2 activity. Sporadic circulation of the pandemic influenza A/H1N1pdm09 was observed in August (week 33) of 2018 but was not observed in 2019 that indicated an irregular nature of the circulation of this virus. Our findings that influenza activities occurred during the rainy season are in agreement with previous studies conducted in several sub-Saharan African countries [8–12, 22–24]. However, overall, it appears that peaks of influenza activity varied across countries in the region.

The circulation of influenza viruses in Guinea is dominated by influenza viruses A/H3N2 and B/Victoria that occurs in an alternate pattern. However, long-term influenza epidemiological and virological surveillance would need to be conducted in order to capture the regularity of the seasonality of influenza virus circulation in Guinea.

## Conclusions

In conclusion, the study provides some insights about influenza viruses that are circulating in Conakry, Guinea and the period of their activities. It shows that influenza B Victoria lineage and influenza A/H3N2 dominated influenza epidemiology and that the two strains differ with regard to their period of activities. This work represents the groundwork for a future epidemiological study on influenza viruses in Guinea.

## Data Availability

The datasets generated and/or analysed during the current study are available from the corresponding authors upon reasonable request.

## References

[1] Iuliano AD (2018) Estimates of global seasonal influenza-associated respiratory mortality: a modelling study. Lancet (London, England) 391, 1285–1300. 10.1016/S0140-6736(17)33293-2 PMC5935243 29248255

[2] Lafond KE (2016) Global role and burden of influenza in pediatric respiratory hospitalizations, 1982–2012: a systematic analysis. PLoS Medicine 13, e1001977. 27011229 10.1371/journal.pmed.1001977 PMC4807087

[3] Nair H (2011) Global burden of respiratory infections due to seasonal influenza in young children: a systematic review and meta-analysis. Lancet (London, England) 378, 1917–1930. 10.1016/S0140-6736(11)61051-9 22078723

[4] World Health Organization (WHO) (2014) Global Epidemiological Influenza Surveillance Standards for Influenza. World Health Organization (WHO).

[5] Ope MO (2011) Risk factors for hospitalized seasonal influenza in rural western Kenya. PLoS One 6, e20111. 21637856 10.1371/journal.pone.0020111 PMC3102693

[6] Cohen C (2013) Severe influenza-associated respiratory infection in high HIV prevalence setting, South Africa, 2009–2011. Emerging Infectious Diseases 19, 1766–1774. 24209781 10.3201/eid1911.130546 PMC3837669

[7] Cohen C (2015) Mortality amongst patients with influenza-associated severe acute respiratory illness, South Africa, 2009–2013. PLoS One 10, e0118884. 25786103 10.1371/journal.pone.0118884 PMC4365037

[8] Niang MN (2012) Sentinel surveillance for influenza in Senegal, 1996–2009. Journal of Infectious Diseases 206(Suppl 1), S129–S135. 10.1093/infdis/jis576 23169958

[9] Bonney JH (2012) Virological surveillance of influenza-like illness among children in Ghana, 2008–2010. Journal of Infectious Diseases 206(Suppl 1), S108–S113. 10.1093/infdis/jis577 23169955

[10] Jones AH (2016) Sentinel surveillance for influenza among severe acute respiratory infection and acute febrile illness inpatients at three hospitals in Ghana. Influenza and other respiratory viruses 10, 367–374. 27239956 10.1111/irv.12397 PMC4947945

[11] Kadjo HA (2013) Sentinel surveillance for influenza and other respiratory viruses in Cote d'Ivoire, 2003–2010. Influenza and Other Respiratory Viruses 7, 296–303. 22863403 10.1111/j.1750-2659.2012.00389.x PMC5779848

[12] Maman I (2014) Implementation of influenza-like illness sentinel surveillance in Togo. BMC Public Health 14, 981. 25239536 10.1186/1471-2458-14-981 PMC4190418

[13] Sagna T (2018) Preliminary results of official influenza and acute respiratory infection surveillance in two towns of Burkina Faso, 2013–2015. BMC Infectious Diseases 18, 330. 30012098 10.1186/s12879-018-3241-3 PMC6048705

[14] Sanou AM (2018) Epidemiology and molecular characterization of influenza viruses in Burkina Faso, sub-Saharan Africa. Influenza and other Respiratory Viruses 12, 490–496. 29350841 10.1111/irv.12539 PMC6005621

[15] Tarnagda Z (2014) Sentinel surveillance of influenza in Burkina Faso: identification of circulating strains during 2010–2012. Influenza and other Respiratory Viruses 8, 524–529. 25074591 10.1111/irv.12259 PMC4181815

[16] (WHO) WHO. Guinea: WHO statistical profile. Available at: https://www.who.int/gho/countries/gin.pdf.

[17] Centers for Disease Control and Prevention (CDC). 2014–2016 Ebola outbreak in West Africa. Available at: https://www.cdc.gov/vhf/ebola/history/2014-2016-outbreak/index.html.

[18] The World Bank. 2014–2015 west Africa Ebola crisis: impact update. Available at: http://www.worldbank.org/en/topic/macroeconomics/publication/2014-2015-west-africa-ebola-crisis-impact-update.

[19] World Health Organisation (WHO) (2015) Protocol for National Influenza Sentinel Surveillance. Brazzaville, Republic of Congo: World Health Organisation (WHO) Regional Office for Africa.

[20] Fitzner J (2018) Revision of clinical case definitions: influenza-like illness and severe acute respiratory infection. Bulletin World Health Organisation 96, 122–128. 10.2471/BLT.17.194514 PMC5791775 29403115

[21] Talla Nzussouo N (2017) Epidemiology of influenza in West Africa after the 2009 influenza A(H1N1) pandemic, 2010–2012. BMC Infectious Diseases 17, 745. 29202715 10.1186/s12879-017-2839-1 PMC5716025

[22] Manirakiza A (2017) Sentinel surveillance of influenza-like illness in the Central African Republic, 2010–2015. Archive Public Health 75, 61. 10.1186/s13690-017-0229-1 PMC5628463 29034093

[23] Nyatanyi T (2012) Influenza sentinel surveillance in Rwanda, 2008–2010. Journal of Infectious Diseases 206(Suppl 1), S74–S79. 10.1093/infdis/jis574 23169976

[24] Wabwire-Mangen F (2016) Epidemiology and surveillance of influenza viruses in Uganda between 2008 and 2014. PLoS One 11, e0164861. 27755572 10.1371/journal.pone.0164861 PMC5068740

